# CD73 Overexpression Promotes Progression and Recurrence of Papillary Thyroid Carcinoma

**DOI:** 10.3390/cancers12103042

**Published:** 2020-10-19

**Authors:** Young Mun Jeong, Haejin Cho, Tae-Min Kim, Yourha Kim, Sora Jeon, Andrey Bychkov, Chan Kwon Jung

**Affiliations:** 1 Department of Hospital Pathology, College of Medicine, The Catholic University of Korea, Seoul 06591, Korea; dydrkf1993@naver.com (Y.M.J.); chuchura@naver.com (Y.K.); thfk38@nate.com (S.J.); 2 Cancer Research Institute, College of Medicine, The Catholic University of Korea, Seoul 06591, Korea; haejincho59@catholic.ac.kr (H.C.); tmkim@catholic.ac.kr (T.-M.K.); 3 Department of Biomedicine & Health Sciences, Graduate School, The Catholic University of Korea, Seoul 06591, Korea; 4 Department of Medical Informatics, College of Medicine, The Catholic University of Korea, Seoul 06591, Korea; 5 Department of Pathology, Kameda Medical Center, Kamogawa, Chiba 296-8602, Japan; bychkov.andrey@kameda.jp; 6 Department of Pathology, Nagasaki University Graduate School of Biomedical Sciences, Nagasaki 852-8523, Japan

**Keywords:** thyroid cancer, CD73, 5′-Nucleotidase, tumor microenvironment, epithelial–mesenchymal transition

## Abstract

**Simple Summary:**

This study aimed to evaluate the clinicopathologic significance of CD73 expression in patients with papillary thyroid carcinoma (PTC) and the potential for CD73 to serve as a therapeutic target of PTC. CD73 was highly expressed in PTC, but not in the normal thyroid tissue. Overexpression of CD73 was associated with unfavorable clinicopathologic characteristics and a shorter recurrence-free survival. The expression level of CD73 mRNA was associated with the abundance of Tregs and dendritic cells, depletion of natural killer (NK) cells, and high expression of immune checkpoint genes and epithelial-to-mesenchymal transition-related genes. CD73 inhibitor attenuated PTC cell proliferation, migration, and invasion in vitro, and suppressed PTC xenograft tumor growth in nude mice. These results suggest that CD73 expression is an unfavorable prognostic marker for patients with PTC. CD73 blockade would be an attractive candidate for therapeutic strategies in patients with advanced PTC.

**Abstract:**

CD73 is involved in tumor immune escape and promotes the growth and progression of cancer cells. The functional role of CD73 expression in papillary thyroid carcinoma (PTC) has not yet been established. In 511 patients with PTC, immunohistochemistry for CD73 on tissue microarrays showed that the high expression of CD73 was associated with an aggressive histologic variant (*p* = 0.002), extrathyroidal extension (*p* < 0.001), lymph node metastasis (*p* < 0.001), and *BRAF*^V600E^ mutation (*p* = 0.015). Survival analysis results showed that patients with high CD73 expression had worse recurrence-free survival (*p* = 0.023). CD73 inhibitors induced G1 cell cycle arrest and apoptosis, inhibited the migration and invasion of PTC cells, and suppressed tumor growth in PTC xenograft nude mice. High expression of CD73 (*NT5E*) mRNA was associated with unfavorable clinicopathologic characteristics, the abundance of Tregs and dendritic cells, depletion of natural killer (NK) cells, and high expression of immune checkpoint genes and epithelial-to-mesenchymal transition-related genes in The Cancer Genome Atlas (TCGA) dataset. Taken together, CD73 expression promotes tumor progression and predicts low recurrence-free survival. Targeting the CD73–adenosine axis in the tumor microenvironment offers an attractive pathway for therapeutic strategies aimed at advanced PTC.

## 1. Introduction

Papillary thyroid carcinoma (PTC) is the most common thyroid cancer, accounting for more than 90% of all cases [1,2]. Most patients with PTC have an excellent prognosis with a 10-year survival rate of more than 90–95% [2,3]. PTC recurrence is, however, a major adverse event after initial treatment, and ranges widely from 1% to 40% depending on the clinicopathological features and molecular signature, particularly when harboring mutation *BRAF*^V600E^ in combination with *TERT* promoter [1]. Although disease recurrence is not immediately life-threatening, it leads to further surgical procedures and radioactive iodine (RAI) ablation, and reduced health-related quality of life for patients [1]. Many studies have investigated how best to identify and manage high-risk patients, but the use of molecular markers is limited [1,4].

Cancer cells communicate with surrounding stromal cells including fibroblasts, lymphatic and vascular endothelial cells, pericytes, adipocytes, and immune cells [5]. The tumor microenvironment consisting of cancer cells and stromal cells induces immune tolerance and tumor angiogenesis, and regulates biological processes such as tumor growth, progression, and evolution [5,6,7,8,9].

Adenosine 5′-triphosphate (ATP) is sequentially dephosphorylated to adenosine diphosphate (ADP) and further to adenosine monophosphate (AMP) by ectonucleoside triphosphate dephosphohydrolase-1 (CD39/ENTPD-1). AMP is then dephosphorylated to adenosine by ecto-5′-nucleotidase (CD73/*NT5E*) [10]. Extracellular adenosine mediates immunosuppressive and anti-inflammatory responses [10]. The CD73/adenosine pathway is involved in immunomodulatory functions and stemness maintenance of mesenchymal stem cells and cancer stem cells [11,12,13]. The CD73/adenosine pathway also operates in the tumor microenvironment. CD73 expression on tumor cells and stromal cells suppresses antitumor responses of immune cells and promotes both tumor growth and cancer progression [10].

CD73 expression is associated with poor prognosis in various cancers including melanoma, colorectal cancer, gastric cancer, gallbladder cancer, and prostate cancer [14,15,16]. In PTC, a recent study reported that CD73 (*NT5E*) mRNA overexpression was associated with lymph node metastasis and tumor size in a small series of cases (*n* = 29), and led the authors to suggest that studies with a larger number of patients are required to clarify their preliminary findings [17]. Currently, it remains elusive as to whether CD73 expression plays a role in tumor progression and prognosis in patients with thyroid cancer.

This study thus aimed to evaluate the prognostic significance of CD73 expression in a large cohort of patients with PTC and to identify the role of CD73 in cancer growth and progression in both in vitro and in vivo models, and further investigated CD73 (*NT5E*) mRNA expression using RNA sequencing data of PTC in The Cancer Genome Atlas (TCGA).

## 2. Results

### 2.1. Baseline Characteristics

The demographic and clinicopathologic features are summarized in Table 1. Histologic variants of 511 PTC cases consisted of 449 classic (including 67 classic PTCs with tall cell features), 17 follicular (including 5 invasive encapsulated follicular and 12 infiltrative follicular variants), 21 tall cell, and 24 other rare variants.

### 2.2. Clinicopathologic Significance of CD73 Expression

**Figure 1:**
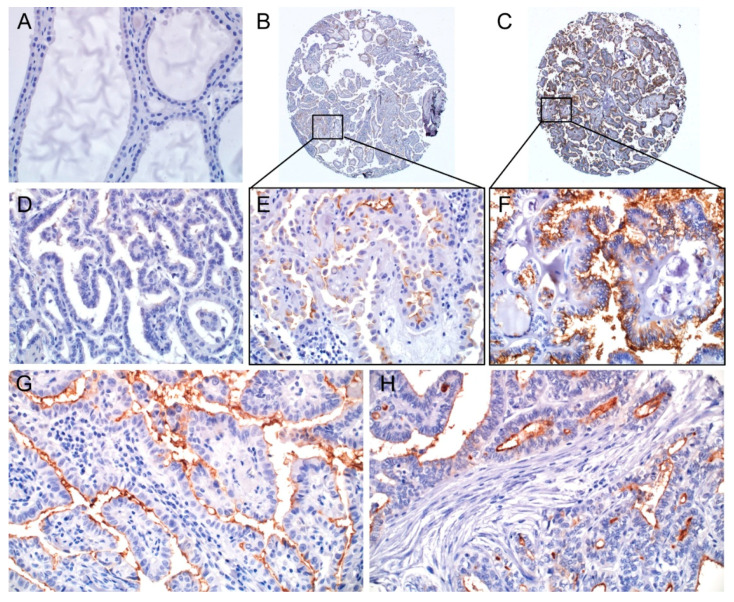
Immunohistochemical expression of CD73 in normal thyroid and papillary thyroid carcinoma (PTC) tumor tissue. (**A**) Normal thyroid tissue is negative for CD73. Tumor cells show positive (**B**,**C**) or negative (**D**) staining for CD73. (**E**) High-powered view of the rectangular area in the (**B**). Tumor cells show apical membranous staining. (**F**) High-powered view of the rectangular area in the (**C**). Tumor cells showed both membranous and cytoplasmic staining. In another CD73 positive case, inflammatory cells (**G**) and fibroblastic and endothelial cells (**H**) in the stroma are negative for CD73, whereas tumor cells are positive. ×400 (**A**,**D**–**H**), ×40 (**B**,**C**).

Normal thyroid follicular cells either adjacent to the tumor or in the control samples did not express CD73. Immunostaining for CD73 was observed in the cell membrane and/or cytoplasm of tumor cells (Figure 1). Membranous staining in cancer cells was considered positive. Inflammatory cells and stromal cells were negative for CD73 (Figure 1G,H).

To investigate the clinicopathologic significance of CD73 expression in PTC, the 511 patients were divided into high (*n* = 370) and low (*n* = 141) expression groups according to a cut-off value of 10% of CD73 immunostained tumor cells (Table 1). High CD73 expression was significantly associated with female gender (*p* = 0.047), aggressive histologic variant (*p* = 0.002), extrathyroidal extension (*p* < 0.001), gross extrathyroidal extension (*p* = 0.010), higher pathologic (p) T category (*p* = 0.024), lymph node metastasis (*p* < 0.001), dyscohesive tumor cells seen at the invasive front (*p* < 0.001), structural disease recurrence (*p* = 0.024), and *BRAF*^V600E^ (*p* = 0.015).

### 2.3. Recurrence-Free Survival

**Figure 2:**
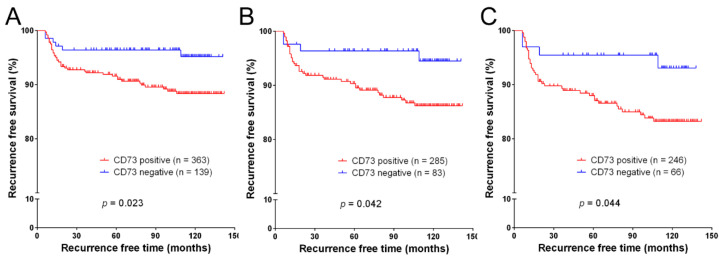
Recurrence-free survival analysis of CD73 expression in patients who underwent thyroid surgery for papillary thyroid carcinoma. (**A**) The recurrence-free survival in 502 patients is significantly correlated with CD73 expression. Stratified survival analyses show a significant association between CD73 expression and recurrence-free survival in 368 patients with extrathyroidal extension (**B**) and in 312 patients with lymph node metastasis (**C**).

Recurrence was defined as structural recurrence detected by imaging modalities and/or cytologic or histologic examinations. Out of 511 patients, 9 were excluded owing to synchronous distant metastasis at the time of surgery (*n* = 3) and less than six months of follow-up data (*n* = 6). During the median follow-up period of 112 months, structural recurrence occurred in 45 (9.0%) of the remaining 502 patients. Kaplan–Meier survival plot and log-rank test showed that high expression of CD73 detected by immunostaining was associated with a worse recurrence-free survival (*p* = 0.023, Figure 2A).

In the stratified survival analyses, high expression of CD73 was also a predictor for decreased recurrence-free survival in subgroups of PTC patients with extrathyroidal extension (*n* = 368, *p* = 0.042, Figure 2B) and lymph node metastasis (*n* = 312, *p* = 0.044, Figure 2C).

On multivariate analysis, lymph node metastasis, gross extrathyroidal extension, and pT category were independently correlated with poor recurrence-free survival (*p* = 0.001, *p* = 0.015, *p* = 0.02, respectively), as shown in Table 2. However, unlike in the univariate analysis, CD73 expression did not reach statistical significance when correlated with recurrence-free survival in multivariate analysis (*p* = 0.085).

### 2.4. Downregulation of CD73 Reduces Migration and Invasion of PTC Cells

**Figure 3:**
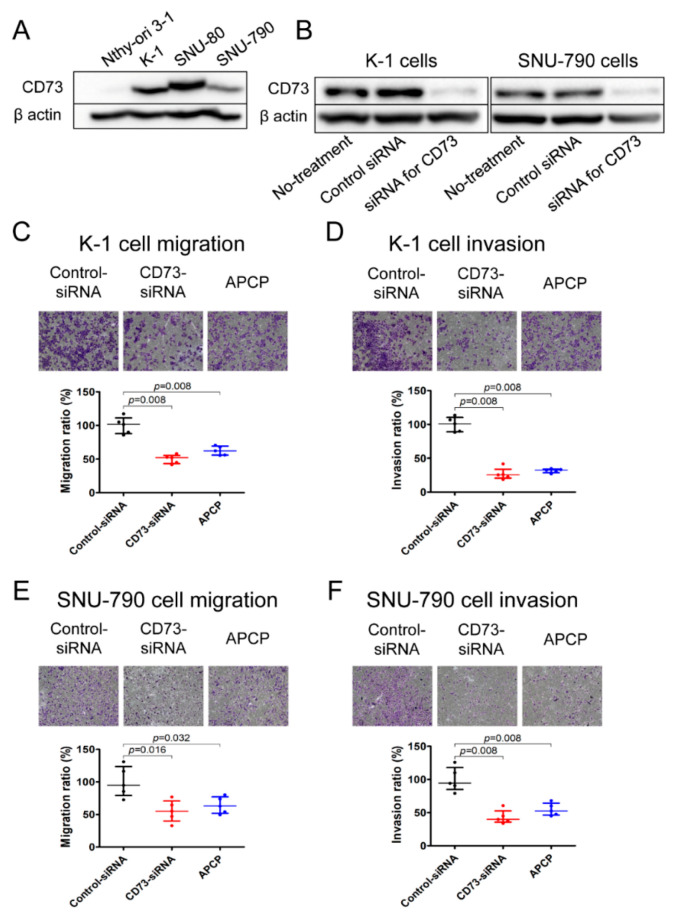
Western blot analysis of CD73 expression and transwell migration and invasion assays in thyroid cell lines. (**A**) Absence of CD73 expression in normal thyroid (Nthy-ori 3-1) and high expression of CD73 in papillary thyroid carcinoma (K-1 and SNU-790) and anaplastic thyroid carcinoma (SNU-80) cell lines. Densitometry data generated for Western blots are shown in Figure S1. (**B**) Inhibition of CD73 expression by siRNA in K-1 and SNU-790 cells. Inhibition of CD73 expression by the administration of CD73-siRNA and specific CD73 inhibitor, adenosine 5′-(α,β-methylene) diphosphate (APCP), downregulates migration and invasion of K-1 (**C**,**D**) and SNU-790 (**E**,**F**) cells. Detailed information can be found in Figure S4. Data are shown as median and interquartile range from five independent experiments. Original magnification, ×100 (**C**–**F**).

We screened the expression levels of CD73 in normal thyroid (Nthy-ori 3-1), PTC (K-1 and SNU-790), and anaplastic thyroid carcinoma (SNU-80) cell lines. CD73 was not expressed in Nthy-ori 3-1 cells, but was highly expressed in both PTC and anaplastic thyroid carcinoma cell lines (Figure 3A).

To assess the effect of CD73 downregulation on PTC cell migration and invasion, K-1 and SNU790 cells were transfected with siRNA for CD73 or treated with specific CD73 inhibitor, adenosine 5′-(α,β-methylene) diphosphate (APCP). CD73 protein expression was significantly lower in cells transfected with CD73-siRNA compared with the control (Figure 3B). Transwells were then used to perform migration and invasion assays.

The migration and invasion of K-1 cells were reduced by 50.0% and 73.1% in CD73-siRNA transfected cells and by 37.4% and 68.4% in APCP treated cells, respectively, compared with the control (Figure 3C,D). The migration and invasion of SNU-790 cells were reduced by 44.8% and 55.4% in CD73-siRNA transfected cells and by 36.9% and 45.2% in APCP treated cells, respectively, compared with the control group (Figure 3E,F). These results show that CD73 plays a role in the migratory and invasive behavior of PTC cells.

### 2.5. Downregulation of CD73 Expression Induces Apoptosis in PTC Cells

To investigate the effect of CD73 on apoptosis in PTC, tumor cells were transfected with CD73-siRNA or treated with APCP, and then the percentage of apoptotic cells was analyzed by R-phycoerythrin-conjugated annexin V (PE) and propidium iodide (PI) double staining and fluorescence-activated cell sorting (FACS).

**Figure 4:**
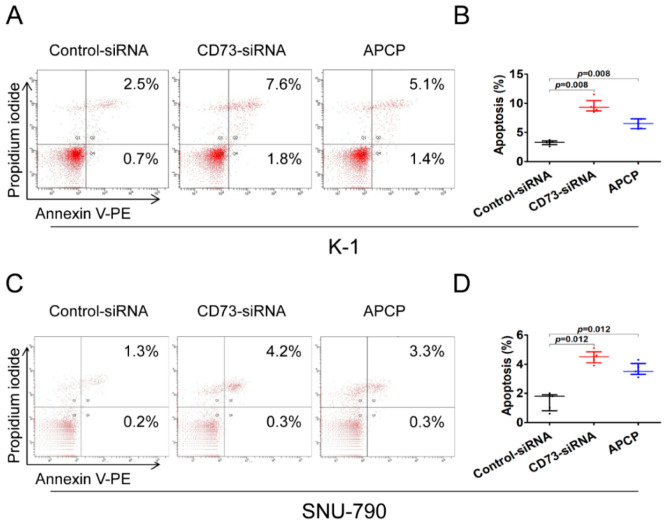
Analysis of apoptosis by flow-cytometry. Downregulation of CD73 expression promotes apoptosis of K-1 cells (**A**,**B**) and SNU-790 cells (**C**,**D**). Downregulation of CD73 was performed using the siRNA for CD73 and adenosine 5′-(α,β-methylene) diphosphate (APCP). Flow cytometry dot plots (**A**,**C**) were divided into four quadrants by the positivity of R-phycoerythrin-conjugated annexin V (PE) and propidium iodide (PI) staining. The lower and upper right quadrants represent early- (annexin V positive and PI negative) and late-stage (annexin V positive and PI positive) apoptotic cells, respectively. The left lower quadrant represents viable cells (double negative cells). The total percentage of apoptotic cells was compared between control and CD73-downregulated cells by siRNA and APCP (**B**,**D**). Data are shown as median and interquartile range from five independent experiments.

In K-1 cells, the percentage of early and late apoptotic cells increased significantly from 3.2% ± 0.2% to 9.4% ± 0.3% (*p* < 0.01) and 6.5% ± 0.3% (*p* < 0.01) in CD73-siRNA and APCP treated groups, respectively (Figure 4A,B). Likewise, the percentage of total apoptotic cells in SNU-790 cell line significantly increased from 1.5% ± 0.6% to 4.5% ± 0.2% (*p* = 0.01) and 3.6% ± 0.3% (*p* = 0.01) in CD73-siRNA and APCP treated groups, respectively (Figure 4C,D). Downregulation of CD73 expression significantly suppressed the proliferation of K-1 and SNU-790 cells (Figure S2).

### 2.6. Downregulation of CD73 Arrests Cell Cycle Progression in PTC Cells

**Figure 5:**
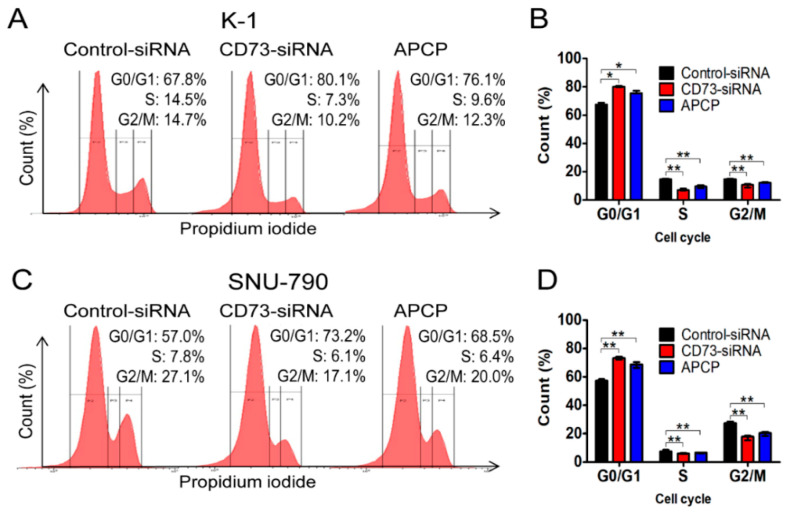
Analysis of cell cycle progression in PTC cell lines by flow cytometry. Downregulation of CD73 arrests cell cycle progression of K-1 cells (**A**,**B**) and SNU-790 cells (**C**,**D**). Data are shown as median and interquartile range from five independent experiments (**B**,**D**). * *p* < 0.05, ** *p* < 0.01 determined by Mann–Whitney test.

To investigate whether a reduction in CD73 influences apoptosis in PTC cells, the cell cycle distribution in K-1 and SNU-790 cells treated with CD73-siRNA and APCP was analyzed using PI staining and FACS (Figure 5).

Knocking-down or inhibiting CD73 by CD73-siRNA or APCP significantly increased the proportion of G0/G1-phase cells in K-1 cells (67.8% ± 0.1% vs. 80.1% ± 0.1% (*p* = 0.01) and 76.1% ± 0.1% (*p* = 0.01), respectively) and in SNU-790 cells (57.0% ± 0.2% vs. 73.2% ± 0.1% (*p* < 0.01) and 68.5% ± 0.3% (*p* < 0.01), respectively) compared with the control group. The percentages of S-phase and G2/M-phase cells were significantly decreased in CD73-downregulated groups of both cell lines. These results suggest that CD73 is involved in the proliferation of PTC cells by inhibiting apoptosis and promoting the cell cycle.

### 2.7. CD73 Inhibition Suppresses Growth of PTC Cell Line-Derived Xenograft in Nude Mice

**Figure 6:**
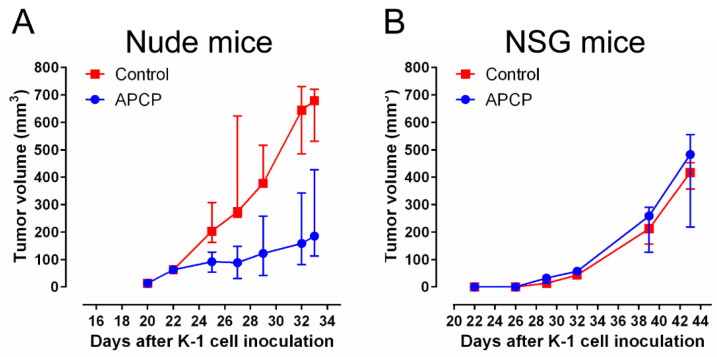
Effects of CD73 inhibitor on the growth of papillary thyroid carcinoma in xenografts. (**A**) K-1 cells (1 × 10^6^) were inoculated subcutaneously into BALB/c nude mice. When tumor size reached 5 mm in diameter, mice were divided into two groups: adenosine 5′-(α,β-methylene) diphosphate (APCP) (*n* = 10) and phosphate-buffered saline (PBS) control (*n* = 10). APCP and PBS were injected into the peritumoral areas twice a week. Data are shown as median and interquartile range. *p*-values at days 25, 27, 29, 32, and 33 were 0.005, <0.001, <0.001, 0.005, and 0.011, respectively. Representative gross specimens are shown in Figure S3. (**B**) To minimize the influence of the immune system, K-1 cells (1 × 10^4^) were inoculated subcutaneously into NOD.Cg-Prkdc^scid^ Il2rg^tm1Wjl^/SzJ (NSG) mice (APCP, *n* = 4; PBS control, *n* = 4).

To investigate the effect of anti-CD73 treatment on tumor growth in vivo, K-1 cells were injected subcutaneously into female BALB/c nude mice. Tumors treated with APCP grew more slowly than phosphate-buffered saline (PBS) treated tumors (Figure 6A). Mean tumor weight at sacrifice was significantly lower in APCP-treated mice than in PBS-treated mice (0.35 ± 0.02 g vs. 0.7 ± 0.02 g, *p* < 0.01).

To investigate whether tumor growth is regulated by CD73 inhibitor in an immune-independent manner, we inoculated K-1 cells into female NOD.Cg-Prkdc^scid^ Il2rg^tm1Wjl^/SzJ (NSG mice), which is one of the most immune deficient strains. There was no significant difference in tumor growth rate between APCP- and PBS-treated groups (Figure 6B).

### 2.8. Clinicopathologic Significance of CD73 (NT5E) mRNA Expression in TCGA Dataset

We analyzed the relationship between CD73 mRNA expression levels and clinicopathologic features as found in the TCGA dataset (Table 3). High CD73 mRNA expression was significantly associated with extrathyroidal extension (*p* < 0.001), advanced pathologic (p) T category (*p* = 0.004), lymph node metastasis (*p* < 0.001), distant metastasis (*p* = 0.025), advanced American Joint Committee on Cancer (AJCC, 7th edition) stage (*p* = 0.002), *BRAF*-like molecular signature (*p* < 0.001), and increased American Thyroid Association (ATA) recurrence risk (*p* < 0.001). However, there was no significant difference in recurrence-free survival between CD73 mRNA high and low expression groups (*p* = 0.657).

### 2.9. Relationship between CD73 (NT5E) mRNA Expression and Tumor Microenvironment in TCGA Dataset

**Figure 7:**
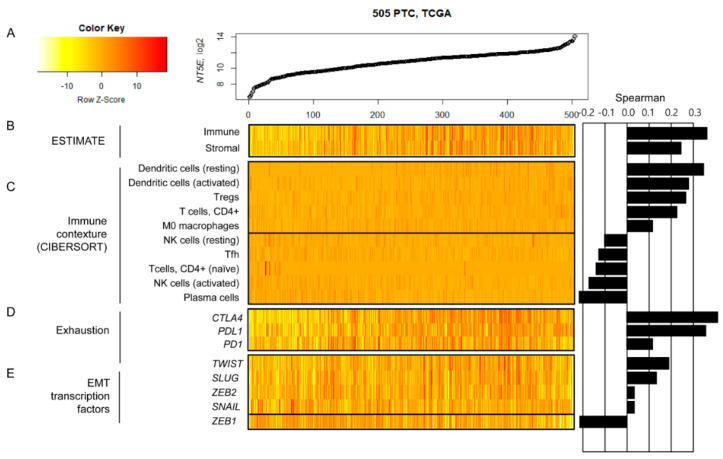
Relationship between CD73 (*NT5E*) mRNA expression and tumor microenvironment-related genes in papillary thyroid carcinoma data from The Cancer Genome Atlas (TCGA). (**A**) A total of 505 PTCs were sorted by their CD73 expression levels. (**B**) The ESTIMATE analysis shows a positive correlation between CD73 transcript levels and both immune and stromal scores. (**C**) In the CIBERSORT analysis, CD73 expression levels were positively correlated with the abundance of dendritic cells and Tregs, but were negatively correlated with transcript levels of natural killer (NK) cells and plasma cells. *NT5E* transcript levels were positively correlated with those of immune checkpoint genes (**D**) and epithelial-to-mesenchymal transformation (EMT) related genes (**E**).

To further dissect the association between transcriptional up-regulation of CD73 (*NT5E*) and unfavorable clinical outcomes in PTC, we performed tumor microenvironment profiling based on RNA-seq data. A total of 505 PTCs in the TCGA data were sorted in order of their CD73 expression (Figure 7A) and various features representing tumor microenvironments in PTC were evaluated. First, the expression-based immune and stromal scores were calculated using the ESTIMATE algorithm (Estimation of STromal and Immune cells in MAlignant Tumor tissues using Expression data), which represents the relative abundance of immune and stromal cells in the individual tumor tissue [18].

A substantial level of correlation between CD73 expression and immune (Spearman’s correlation = 0.393) and stromal (Spearman’s correlation = 0.393) scores was observed (Figure 7B). This indicates that PTC with high levels of CD73 expression are highly infiltrated with both immune and stromal cells.

Next, we used the CIBERSORT algorithm (Cell-type Identification by Estimating Relative Subsets of RNA Transcripts) to estimate the relative abundance of 22 immune cell types [19]. We selected ten immune cells whose estimated abundances were significantly correlated with CD73 transcript levels (adjusted *p*-values < 0.05, Figure 7C). We observed that the abundance of dendritic cells and regulatory T cells (Tregs) is positively correlated with CD73 transcript levels, while that of natural killer (NK) cells and plasma cells is inversely correlated. Tregs and NK cells are immune cell components that may function in favor of, or against, the proliferation of cancer cells, respectively, suggesting that up-regulation of CD73 transcription is associated with the acquisition of immune contexture, supporting the progression of PTCs.

Cytotoxic T lymphocytes (CTLs) have a strong positive impact on patients’ survival for various types of tumors. We observed that the expression of immune checkpoint genes *CTLA4*, *PDL1*, and *PD1* was positively correlated with CD73 transcript levels, reflecting the exhausted phenotypes of infiltrated CTLs in PTCs with CD73 expression (Figure 7D), although CD8+ T lymphocytes did not show a substantial correlation with CD73 transcript levels.

The high level of stromal cell infiltration is suggestive of the activation of epithelial-to-mesenchymal transformation (EMT). Among the five transcription factors regulating EMT, four (*TWIST*, *SLUG*, *ZEB2*, and *SNAIL*) were positively correlated with CD73 transcript levels, suggesting that PTCs with high CD73 expression may activate EMT (Figure 7E).

## 3. Discussion

High expression of CD73 protein by cancer cells was associated with poor clinicopathologic features including extrathyroidal extension, lymph node metastasis, *BRAF*^V600E^ mutation, and worse recurrence-free survival in patients with PTC. Treatment with APCP, a CD73 inhibitor, suppressed tumor growth in the PTC xenograft mouse model.

Although most studies have reported that overexpression of CD73 is associated with poor prognosis in a wide range of different cancers [14,15,16], several studies have shown a relationship with favorable prognosis in lung and gastric cancers [20]. There has been a paucity of studies investigating the effect of CD73 expression on clinicopathologic outcomes in patients with thyroid cancers. A recent study revealed that high expression of CD73 mRNA was associated with larger tumor size and lymph node metastasis, but CD73 protein expression by immunohistochemistry did not reach statistical significance [17]. These conflicting findings between mRNA and protein expression might be related to the small sample size in which the case number was less than 30. In contrast, to provide greater clarity, we investigated expression levels of CD73 protein in a large study cohort (*n* = 511) and observed a significant association between high CD73 expression and unfavorable clinicopathologic characteristics. Furthermore, survival analysis found that patients with CD73 overexpression had an increased risk of structural recurrence. We also revealed a significant relationship between high expression of CD73 mRNA and poor clinicopathologic features in the TCGA study cohort. 

Migration and invasion of cancer cells are the initial step in metastasis and dissemination from primary tumors [21]. We found that PTC cells overexpressed CD73 and CD73 inhibition suppressed cancer cell migration and invasion in transwell assays. In our clinical samples and in the TCGA database, the expression of CD73 protein and CD73 mRNA was associated with lymph node metastasis and extrathyroidal extension. These findings suggest that CD73 is involved in the dissemination of PTC to adjacent soft tissue and lymph nodes.

CD73 converts extracellular AMP to adenosine. The converted adenosine has immunosuppressive functions, promotes the expression of cancer stemness and EMT-related genes in solid tumors [13,22], and inhibits the functions of tumor-infiltrating immune cells, consequently leading to tumor progression [10]. Stimulation of the adenosine receptor on immune cells upregulates Treg inhibitory activity; inhibits the function and expansion of myeloid-derived suppressor cells, NK cells, and M1 macrophages; and induces M2 macrophage polarization within the tumor microenvironment [23]. Dendritic cells are a heterogenous cell population containing different subsets [24]. CD73-derived adenosine in the tumor microenvironment causes specific differentiation of dendritic cells that have immunosuppressive and immune tolerance properties and promote tumor growth [25]. 

In the present study, CD73 mRNA expression was associated with enrichment of Tregs and dendritic cells along with a depletion of NK cells in the immune contexture. The transcriptional upregulation of immune checkpoint genes (*CTLA4*, *PDL1*, and *PD1*) suggests the exhaustive status of T cells as well as potential resistance to immune checkpoint inhibitors for CD73-upregulated tumors. These results support the hypothesis that CD73 expression by tumor cells suppresses antitumor immunity in the PTC microenvironment. 

In the tumor microenvironment, fibroblasts, endothelial cells, and Tregs can express CD73 and enhance antitumor immunity [26]. NK cells expressing CD73 can undergo phenotypic changes that acquire the expression of immune checkpoint receptors and contribute to tumor immune escape [27]. Therefore, our study does not exclude the possibility that CD73 expression by the PTC microenvironment may play role in antitumor immunity, although inflammatory cells and stromal cells were negative for CD73 by immunohistochemistry. Further studies will be necessary to verify whether PTC-associated stromal cells including immune cells, fibroblasts, and endothelial cells express CD73 and modulate tumor immunity.

EMT is associated with progression and metastasis of PTC [28]. Previous studies showed that CD73 is a key regulator for the maintenance of stem-like characteristics in mesenchymal stromal/stem cells and cancer cells [13,22]. When we analyzed the TCGA dataset, CD73 (*NT5E*) mRNA expression was associated with the expression of EMT-related genes (*TWIST*, *SLUG*, *ZEB2*, and *SNAIL*). These data suggest that CD73 expression in PTC may play an important role in the activation of EMT during cancer growth and progression. 

As CD73 expression is associated with cancer progression, poor clinical outcomes, and resistance to chemotherapy [14,15,16], targeting the CD73–adenosine axis in the tumor microenvironment has been actively studied as an attractive therapeutic strategy to inhibit tumor progression and improve cancer immunotherapy [29]. Clinical trials of anti-CD73 monoclonal antibodies, CD73 inhibitors, and adenosine receptor antagonists listed in the National Cancer Institute Drug Dictionary (www.cancer.gov/publications/dictionaries/cancer-drug/) are underway in patients with advanced solid cancers. These agents inhibit the conversion of extracellular AMP to adenosine and consequently suppress the adenosine-mediated inhibitory effect on the immune system against cancer cells. A number of studies have reported that CD73–adenosine targeting agents can inhibit tumor progression and angiogenesis, and prevent metastasis in solid tumors of animal models [23]. CD73 expression was also associated with a poor immune response to PD-1/PD-L1 inhibitors and blockade of CD73 enhanced the effect of anti-CTLA-4 and PD-1 inhibitors [20].

The expression of CD73 in tumor cells can be regulated by microRNAs [30]. A recent study showed a negative correlation between the expression of CD73 mRNA and miR-422a in head and neck squamous cell carcinoma [31]. The inhibition of endogenous miR-422a increased the expression level of the CD73 protein. Reduced levels of miR-422a were associated with shorter relapse-free survival time in head and neck squamous cell carcinoma [31]. In colorectal cancer, miR-30a and miR-187 directly regulate the expression level of CD73 mRNA and protein and play roles in suppressing tumor growth and progression [32,33]. Transfection of miR-187 inhibited colorectal cancer cell proliferation and migration [33]. These findings suggest that microRNAs targeting CD73 may serve as a therapeutic strategy for the treatment of cancer patients.

We found that a CD73 inhibitor induced apoptosis and arrested cell cycle progression in vitro, prompting us to further examine whether the inhibitor could inhibit PTC growth in an immune-independent manner. Tumor growth was significantly suppressed by APCP treatment in BALB/c nude mice, but not in NSG mice. Given that nude mice are missing both thymus and T cells, whereas super-immunodeficient NSG mice lack T cells, B cells, and NK cells, CD73 inhibitors may affect both CD73-expressing PTC cells and the greater tumor immune environment, thus promoting antitumor immunity in the PTC microenvironment. Therefore, CD73 targeting agents as a novel therapeutic approach may offer additive or synergistic therapeutic benefits to patients with advanced PTCs, and merits further experimental investigation. However, targeting CD73 alone may have limited inhibition effects.

## 4. Materials and Methods

### 4.1. Patients

This study was approved by the Institutional Review Committee of the Catholic University of Korea Seoul St. Mary’s Hospital (KC16SISI0104 and KC16SISI0709). Written informed consent was obtained from all patients. We enrolled 511 patients who underwent surgery for PTC from January 2008 to December 2010. The present study was performed on the same cohort used in our previous studies [34,35,36,37]. However, the number of patients was different because tissue loss inevitably occurs during sectioning and staining of the same archival tissue blocks. Clinical follow-up data were updated as of March 2020.

To purify the study cohort, we excluded indolent and high-grade tumors, which could alter the results of survival analysis. The former included papillary microcarcinomas less than 1 cm in diameter and non-invasive follicular thyroid neoplasm with papillary like nuclear features (NIFTP), and the latter were PTCs with tumor necrosis or significant mitotic activity. The PTC variants were determined according to the 4th edition of the World Health Organization classification of tumors of endocrine organs [38]. PTC was defined as a tall cell variant if it contained 30% or more tall cells. A tumor consisting of less than 30% of tall cells was defined as a classic PTC with tall cell features. Subtypes of PTC comprised of classic PTC (*n* = 382); classic PTC with tall cell features (*n* = 67); infiltrative follicular variant (*n* = 12); invasive encapsulated follicular variant (*n* = 5); tall cell variant (*n* = 21); and other variants (*n* = 24) including oncocytic (*n* = 10), Warthin-like (*n* = 9), hobnail (*n* = 2), diffuse sclerosing (*n* = 1), solid (*n* = 1), and cribriform-morular (*n* = 1). Classic PTCs with tall cell features were grouped into classic PTCs for statistical analysis.

Cancer staging was determined according to the 8th edition of the AJCC cancer staging manual [2]. All patients were regularly followed every 6–12 months using thyroid function tests and at least a yearly neck ultrasound. Recurrence was defined as structural recurrence confirmed by radiological, histological, or cytologic examination. Recurrence-free survival was defined as the time from 6 months after initial surgery to structural recurrence of the tumor. Any incomplete biochemical or structural response detected within 6 months after initial surgery was considered persistent disease, reflecting incomplete surgery.

### 4.2. Tissue Microarray

Tissue microarrays (TMAs) were designed in a 5 × 10 subarray with 2 mm cores at a 1 mm spacing. Each TMA block contained 47 cases of PTC and control tissue cores that consisted of normal thyroid, tonsil, and placental tissue. TMAs were cut into 4 µm thick sections and used for immunohistochemistry.

### 4.3. Immunohistochemistry

Tissue sections were incubated at 60 °C for 1 h; deparaffinized with xylene; and then rehydrated with 100%, 95%, and 70% ethanol. Antigen retrieval was performed with sodium citrate buffer (pH 6.0) in a pressure cooker for 20 min. The activity of endogenous peroxidase was blocked with 3% hydrogen peroxide in methanol for 15 min at room temperature. The slides were incubated with anti-human CD73 rabbit monoclonal antibody (1:200, clone D7F9A, Cell Signaling Technology, Danvers, MA, USA; No. 13160) in a humidified chamber for 1 hour at room temperature. Immunoreactivity of CD73 was visualized using a Polink-1 HRP Broad DAB kit (GBI Labs, Mukilteo, WA, USA; No. D11-18) and then counterstained with Harris’s hematoxylin.

The CD73 immunostaining was evaluated independently by three investigators (Young Mun Jeong, Yourha Kim, and Chan Kwon Jung) in a blinded manner. Consistent with previous publications, a membranous immunostaining of ≥10% of tumor cells was defined as high CD73 expression and <10% membranous immunostaining of tumor cells was considered as low expression [39]. The placental tissue was used as a positive control. A consensus was reached when there was discrepancy between observers.

### 4.4. BRAF Sanger Sequencing

Total genomic DNA was extracted from manually dissected whole tissue sections of paraffin-embedded tissue blocks using RecoverAll™ Total Nucleic Acid Isolation Kit (Invitrogen, Carlsbad, CA, USA; No. AM1975). PCR reaction was performed using *BRAF* exon 15-specific primers (forward 5′-TCATAATGCTTGCTCTGATAGGA-3′ and reverse 5′-GGCCAAAAATTTAATCAGTGGA-3′). Bidirectional sequencing was performed using the same primers and BigDye Terminator sequencing kit (Applied Biosystems, Foster City, CA, USA; No.4337457 on a 3730xl DNA analyzer (Applied Biosystems)), as previously described [34,36,40].

### 4.5. Cell Lines and Cell Culture

The PTC cell lines, K-1, and SNU-790 cells were purchased from the European Collection of Cell Cultures (Salisbury, Wiltshire, UK) and Korean Cell Line Bank (Seoul National University, Seoul, Korea), respectively. K-1 cells were cultured in Dulbecco’s modified Eagle’s Medium/Nutrient Mixture F-12 (DMEM/F12, Gibco, Grand Island, NY, USA; No. 11330-032) supplemented with inactivated 10% fetal bovine serum (Hyclone, GE Healthcare, Logan, UT, USA; No. SH30084.03), 100 U/mL penicillin, and 100 µg/mL streptomycin (Gibco; No. 15140-122). SNU-790 cells were cultured in Roswell Park Memorial Institute 1640 medium (RPMI-1640, Gibco; No. 22400-089) supplemented with inactivated 10% fetal bovine serum, 100 U/mL penicillin, 100 µg/mL streptomycin (Gibco; No. 15140-122), and GlutaMax-1 (Gibco; No. 35050-061). Cells were maintained in a humidified incubator at 37 °C with 5% CO_2_.

### 4.6. Western Blot Analysis

Cells were washed three times with PBS and lysed with radioimmunoprecipitation assay (RIPA) buffer (Thermo Fisher Scientific, Waltham, MA, USA; No. 89900). The protein concentration of the dissolved cells was quantified by a Pierce Bicinchoninic Acid (BCA) protein assay kit (Thermo Fisher Scientific; No. 23227). The proteins were separated by 10% sodium dodecyl sulphate–polyacrylamide gel electrophoresis (SDS-PAGE), and subsequently transferred onto polyvinylidene difluoride membrane (Merck Millipore, Burlington, MA, USA; No. IPVH00010). Membranes were incubated with blocking buffer (Thermo Fisher Scientific; No. 37543) for 1 hour at room temperature and then with the anti-human CD73 monoclonal rabbit antibody (1:1000, Cell Signaling Technology; No. 131605) for 16 hours at 4 °C. The membrane was washed with Tris-buffered saline and incubated with goat anti-rabbit IgG horseradish peroxidase linked antibody (1:2500, Cell Signaling Technology; No. 7074S) for 1 hour at room temperature. Proteins were detected using a SuperSignal West Pico Chemiluminescent Substrate kit (Thermo Fisher Scientific; No. 34578) and quantified using the PXi Touch Imaging System (Syngene, Frederick, MD, USA). The protein levels were normalized to β-actin.

### 4.7. CD73 Inhibitor and siRNA

K-1 and SNU-790 cells (2 × 10^5^) were seeded onto 60 mm dishes and incubated in antibiotic-free culture medium for 24 h at 37 °C. The cell culture medium was replaced with antibiotic-free media containing 100 µM APCP, a CD73 inhibitor (Sigma-Aldrich, St. Louis, MO, USA; No. M3763), and cultured for 24 h (K-1 cells) and 48 h (SNU-790 cells), respectively.

CD73 knockdown was conducted using a CD73 siRNA (Dharmacon, Lafayette, CO, USA; No. D-008217-01) according to the manufacturer’s instructions. The target sequence of the siRNA used was as follows: CD73-siRNA, 5′-GAACCUGGCUGCUGUAUUG-3′, and that of non-targeting siRNA was 5′-UGGUUUACAUGUCGACUAA-3′. The cells were transfected with 20 nM non-targeting siRNA or CD73 siRNA using Lipofectamine 2000 (Invitrogen, Carlsbad, CA, USA; No. 11668-019).

### 4.8. Transwell Invasion and Migration Assays

The invasion assay was performed using a 24-well Transwell chamber with a pore size of 8 µm (Corning, Corning, NY, USA; No. 353097). The insert was coated with 50 µL Matrigel (1:15 dilution, Corning; No. 354230) and the experiments proceeded without antibiotics in the media. Cells were harvested after transfection or inhibitor treatment for 24 h and 200 µL of no serum media containing K-1 cells (2 × 10^4^) or SNU-790 cells (2 × 10^4^) was transferred to the Matrigel-coated membranes. The lower chamber was filled with medium (600 µL) containing 15% fetal bovine serum (FBS). Following incubation for 24 h at 37 °C, the remaining cells on the upper surface of the membrane were removed. Methanol was used to fix the cells on the lower surface for 15 min, and stained with crystal violet (0.1%). Images of stained cells were captured using a digital camera connected to a microscope (EVOS XL Core, Thermo Fisher Scientific). Images were taken from five randomly selected microscopic fields at high magnification (400×). Cell quantitation was performed by processing all obtained images using ImageJ software (http://imagej.nih.gov/ij/). 

The migration assay proceeded identically except for the Matrigel layer, and K-1 cells (8 × 10^3^) or SNU-790 cells (1.5 × 10^4^) were added to the upper chamber. Each experiment was repeated five times.

### 4.9. Flow Cytometry for the Analysis of Apoptosis

Cells were harvested, washed with cold PBS, suspended in 100 µL annexin V binding buffer (BD Biosciences, San Diego, CA, USA; No. 556454), and then stained with 1 µL propidium iodide (0.1 mg/mL, Sigma-Aldrich, St. Louis, MO, USA; No. P4864) and 1 µL annexin V-PE (BD Biosciences; No. 556422). After incubation for 15 min at room temperature in the dark, cells were washed with 400 µL annexin V binding buffer and then immediately analyzed using a flow cytometer (BD FACSCanto I, BD Biosciences) and BD FACSDiva software. Each experiment was repeated five times.

### 4.10. Flow Cytometry Analysis for Cell Cycle

Cells were harvested, washed with cold PBS, and fixed in 1 mL of 70% cold ethanol in the dark for 30 min at 4 °C, and then washed with cold PBS. The cells were suspended in 500 µL PBS containing RNase A (0.2 mg/mL) for 30 min at 37 °C in the dark. Cells were stained with propidium iodide (0.05 mg/mL) for 20 min at 4 °C in the dark and then analyzed using a flow cytometer (BD FACSCanto I, BD Biosciences) and BD FACSDiva software. Each experiment was performed in pentaplicate.

### 4.11. Xenograft Mice

K-1 cells at 1 × 10^6^/injection were inoculated subcutaneously into 7-week-old female BALB/c nude mice (Orientbio, Seong-nam, Korea). When the tumor size reached a size of 5 mm in diameter, the mice were divided into PBS-treated control (*n* = 10) and APCP treated (*n* = 10) groups randomly. PBS and CD73 inhibitor APCP (400 µg/mouse) were injected into the peritumoral soft tissue of the two groups twice a week, respectively. The volume of tumors was measured with Vernier calipers twice a week according to the following formula: a × b^2^ × 0.5 (a, maximum diameter; b, vertical diameter). Mice were sacrificed when tumor size reached a threshold size of 10 mm diameter.

To minimize the influence of immune cells on the APCP treatment, we repeated the experiment using female NOD.Cg-Prkdc^scid^ Il2rg^tm1Wjl^/SzJ mice (NSG mice, Jackson Laboratory, Bar Harbor, ME, USA).

The Institutional Animal Care and Use Committee of The Catholic University of Korea approved all animal protocols (No. 2019-0141-02). All experiments were performed in accordance with the Laboratory Animals Welfare Act, the Guide for the Care and Use of Laboratory Animals, and the Guidelines and Policies for Rodent experiment of our institution with the earned accreditation of the Korea Excellence Animal laboratory Facility from Korea Food and Drug Administration, and the Association for Assessment and Accreditation of Laboratory Animal Care (AAALAC) International.

### 4.12. The Cancer Genome Atlas (TCGA) Data Analysis

We downloaded RNA-Seq mRNA expression data normalized by expectation maximization (RSEM) for PTC (*n* = 505), from the NCI’s Genomic Data Commons (GDC) portal (https://gdc.cancer.gov). The clinicopathological and molecular data were obtained from the index article on thyroid carcinoma by the TCGA network [41]. The expression levels of CD73 (*NT5E*) mRNA were grouped into high (≥median) and low (<median) expression based on the median value of mRNA expression.

Cancer staging in the TCGA data was based on the 7th edition of the AJCC cancer staging system [42]. The degree of extrathyroidal extension was classified into minimally (T3), moderately (T4a), or very (T4b) advanced invasion.

We estimated the score representing the proportion of infiltrating immune and stromal cells in the PTC using ESTIMATE R packages [18], as described previously [37,43]. The relative abundance of infiltrating immune cells in the PTC was analyzed using the support vector regression-based CIBERSORT algorithm [19], as we have described previously [37,43].

### 4.13. Statistical Analysis

The relationship between clinicopathologic features and CD73 expression was analyzed using a Pearson chi-square or Fisher’s exact for categorical variables, when appropriate. In vivo and xenograft experimental data were obtained from five independent experiments. Data are shown as median and interquartile range and analyzed by the Mann–Whitney U test. The correlation between *NT5E* mRNA and other gene expression values was analyzed by the non-parametric Spearman’s rank correlation.

All statistical values were calculated using the statistical software program SPSS (version 21.0, IBM Corp, Armonk, NY, USA), GraphPad Prism (version 6.07, La Jolla, CA, USA), and R software (version 3.5.3). *p*-value < 0.05 was considered statistically significant.

## 5. Conclusions

We show that CD73 expression is associated with PTC progression and recurrence. Suppression of CD73 expression induces apoptosis of PTC cells and inhibits migration and invasion of PTC cells and tumor growth in xenografts. Therefore, modulation of the CD73–adenosine axis in the tumor microenvironment might represent an attractive therapeutic target for advanced PTC.

## Figures and Tables

**Table 1:** Relationship between CD73 expression and clinicopathologic features in 511 patients with papillary thyroid carcinoma.

Characteristic	*n*	CD73 Expression		*p*-Value
		High (*n* = 370)	Low (*n* = 141)	
**Age (years)**	**511**			0.436
<55	364	260 (71.4%)	104 (25.6%)	
≥55	147	110 (74.8%)	37 (25.2%)	
**Sex**	**511**			0.047
Female	403	300 (74.4%)	103 (25.6%)	
Male	108	70 (64.8%)	38 (35.2%)	
**Histologic variant**	**511**			<0.001
Classic	449	323 (71.9%)	126 (28.1%)	
Follicular variant	17	3 (17.6%)	14 (82.4%)	
Tall cell variant	21	21 (100.0%)	0 (0.0%)	
Other	24	23 (95.8%)	1 (4.2%)	
**Histologically aggressive variant ^1^**	**511**			0.002
Aggressive variant	23	23 (100.0%)	0 (0.0%)	
Non-aggressive variant	488	347 (71.1%)	141 (28.9%)	
**Extrathyroidal extension ^2^**	**511**			<0.001
Present	376	292 (77.7%)	84 (22.3%)	
Absent	135	78 (57.8%)	57 (42.2%)	
**Gross extrathyroidal extension**	**511**			0.010
Present	99	82 (82.8%)	17 (17.2%)	
Absent	412	288 (69.9%)	124 (30.1%)	
**Pathologic (p) T category**	**511**			0.024
pT1-2	409	287 (70.2%)	122 (29.8%)	
pT3-4	102	83 (81.4%)	19 (18.6%)	
**pN category**	**511**			<0.001
pN0	195	120 (61.5%)	75 (38.5%)	
pN1a	211	165 (78.2%)	46 (21.8%)	
pNb	105	85 (81.0%)	20 (19.0%)	
**Dyscohesive cells ^3^**	**511**			<0.001
Present	346	276 (79.8%)	70 (20.2%)	
Absent	165	94 (57.0%)	71 (43.0%)	
**AJCC stage, 8th edition**	**511**			0.162
Stage 1	419	298 (71.7%)	121 (28.9%)	
Stage 2	79	61 (77.2%)	18 (22.8%)	
Stage 3	9	8 (88.9%)	1 (11.1%)	
Stage 4	4	3 (75.0%)	1 (25.0%)	
**Structural recurrence ^4^**	**511**			0.024
Present	45	39 (86.7%)	6 (13.3%)	
Absent	457	324 (70.9%)	133 (29.1%)	
***BRAF*** **^V600E^**	**511**			0.015
Present	428	319 (74.5%)	109 (25.5%)	
Absent	83	51 (61.4%)	32 (38.6%)	

^1^ Aggressive variant included tall cell variant (*n* = 21), hobnail variant (*n* = 2), and solid variant (*n* = 1). ^2^ Extrathyroidal extension included minimal (*n* = 277) and gross (*n* = 99) extrathyroidal extension. ^3^ Dyscohesive cells were evaluated at the invasive fronts of papillary thyroid carcinoma. ^4^ Structural recurrence was analyzed in 502 patients with available follow-up. AJCC, American Joint Committee on Cancer.

**Table 2:** Univariate and multivariate Cox regression analysis of prognostic variables for recurrence-free survival in 502 patients with papillary thyroid carcinoma.

Variable	Univariate Analysis		Multivariate Analysis	
	HR (95% CI)	*p*-Value	Adjusted HR (95% CI)	*p*-Value
**Age (years)**				
<55	1		1	
≥55	1.357 (0.672–2.741)	0.395	0.700 (0.321–1.524)	0.369
**Sex**				
Female	1		1	
Male	1.347 (0.696–2.609)	0.377	1.186 (0.606–2.320)	0.619
**Histologic subtype**				
Non-aggressive	1		1	
Aggressive	0.586 (0.182–1.891)	0.371	1.161 (0.352–3.824)	0.807
**Gross extrathyroidal extension**				
Absent	1		1	
Present	1.964 (1.045–3.691)	0.036	6.489 (1.441–29.217)	0.015
**Pathologic (p) T category**				
pT1-2	1		1	
pT3-4	2.308 (1.254–4.250)	0.007	10.022 (2.342–42.890)	0.002
**Lymph node metastasis**				
Absent	1		1	
Present	8.995 (2.788–29.021)	<0.001	7.480 (2.299–24.343)	0.001
**CD73 expression**				
Low	1		1	
High	2.603 (1.102–6.150)	0.029	2.158 (0.899–5.177)	0.085

Aggressive histologic subtype includes 21 tall cell variants, 2 hobnail variants, and 1 solid variant. HR, hazard ratio; CI: confidence interval.

**Table 3:** Correlation between CD73 (*NT5E*) mRNA expression and clinicopathologic features of papillary thyroid carcinoma in The Cancer Genome Atlas (TCGA) database.

Characteristic	*n*	CD73 (*NT5E*) mRNA		*p*-Value
		High Expression (*n* = 223)	Low Expression (*n* = 231)	
**Age (years)**	**454**			0.116
<45	211	112(53.1%)	99(46.9%)	
≥45	243	111(45.7%)	132(54.3%)	
**Sex**	**454**			0.471
Female	123	57(46.3%)	66(53.7%)	
Male	331	166(50.2%)	165(49.8%)	
**Histologic variant**	**454**			0.489
Classic	312	172(55.1%)	140(44.9%)	
Follicular	99	18(18.2%)	81(81.8%)	
Tallcell	34	27(79.4%)	7(20.6%)	
Other	9	6(66.7%)	3(33.3%)	
**Extrathyroidal extension**	**441**			<0.001
None	309	130(42.1%)	179(57.9%)	
Minimal(pT3)	117	80(68.4%)	37(31.6%)	
Moderate/advanced(pT4)	15	8(53.3%)	7(46.7%)	
**Pathologic (p) Tcategory**	**452**			0.004
pT1	131	52(39.7%)	79(60.3%)	
pT2	152	69(45.4%)	83(54.6%)	
pT3	151	91(60.3%)	60(39.7%)	
pT4	18	10(55.6%)	8(44.4%)	
**pN category**	**454**			<0.001
pN0	208	85(40.9%)	123(59.1%)	
pN1	201	122(60.7%)	79(39.3%)	
pNX	45	16(35.6%)	29(64.4%)	
**Distant metastasis**	**453**			0.025
M0	242	130(53.7%)	112(46.3%)	
M1	8	5(62.5%)	3(37.5%)	
MX	203	88(43.3%)	115(56.7%)	
**AJCC stage, 7th edition**	**452**			0.002
Stage I	260	125(48.1%)	135(51.9%)	
Stage II	49	14(28.6%)	35(71.4%)	
Stage III	98	53(54.1%)	45(45.9%)	
Stage IV	45	30(66.7%)	15(33.3%)	
***BRAF*-*RAS* signature**	**391**			<0.001
*BRAF*-like	272	183(67.3%)	89(32.7%)	
*RAS*-like	119	14(11.8%)	105(88.2%)	
**ATA recurrence risk group**	**442**			<0.001
Low	166	56(33.7%)	110(66.3%)	
Intermediate	252	146(57.9%)	106(42.1%)	
High	24	13(54.2%)	11(45.8%)	

AJCC, American Joint Committee on Cancer; ATA, American Thyroid Association. The AJCC 7th edition cancer staging was used in the TCGA dataset.

## Supplementary Materials

The following are available online at https://www.mdpi.com/2072-6694/12/10/3042/s1, Figure S1: Densitometric analysis of CD73 expression on Western blot assays shown in Figure 3, Figure S2: Downregulation of CD73 expression inhibits cell proliferation, Figure S3: Photographs of gross specimens of tumors in BALB/c nude mice shown in Figure 6A, Figure S4: Detail information about Figure 3 and Figure S1.
