# Supplementary material for: CD73 Overexpression Promotes Progression and Recurrence of Papillary Thyroid Carcinoma
Source: Cancers (Basel). 2020 Oct 19;12(10):3042. doi: 10.3390/cancers12103042 (PMC7603384; doi:10.3390/cancers12103042)
Supplement: Supplementary file 1 [file cancers-12-03042-s001.pdf]

Supplementary Materials

CD73 Overexpression Promotes Progression and Recurrence of Papillary Thyroid Carcinoma

Young Mun Jeong, Haejin Cho, Tae-Min Kim, Yourha Kim, Sora Jeon, Andrey Bychkov and Chan Kwon Jung

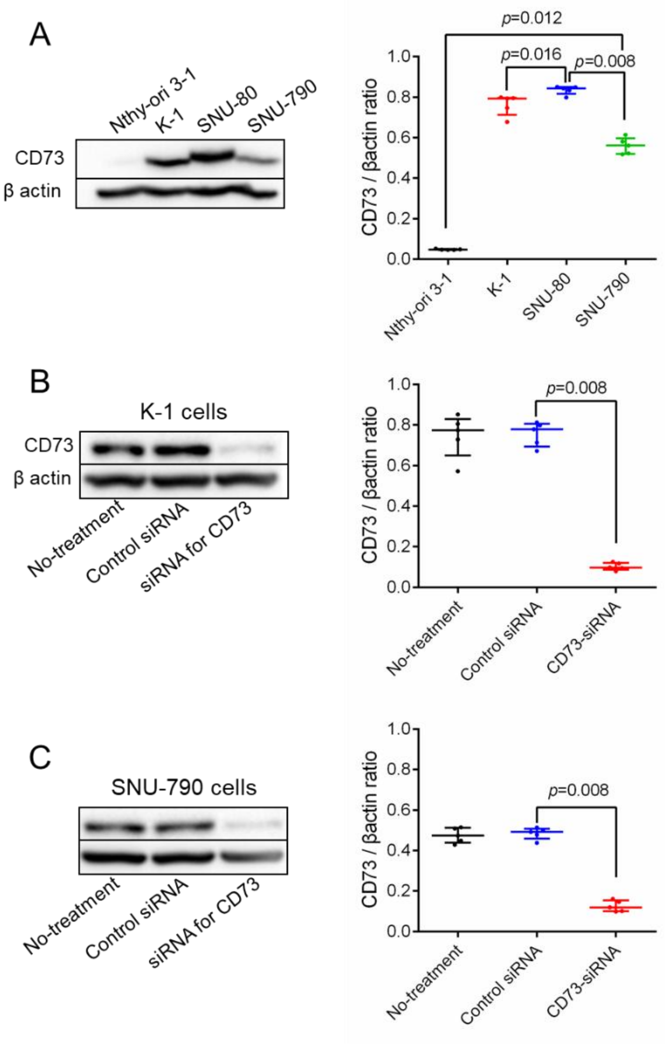

Figure S1. Densitometric analysis of CD73 expression on Western blot assays shown in Figure 3. (**A**) The expression of CD73 expression in papillary thyroid carcinoma (K-1 and SNU-790) and anaplastic thyroid carcinoma (SNU-80) cell line compared to that in a normal thyroid (Nthy-ori 3-1). (**B** and **C**) Inhibition of CD73 expression by siRNA in K-1 and SNU-790 cells. The intensities of protein bands were calculated using GeneTools Analysis Software (version 3.7, Syngene, Cambridge, UK). Data are shown as median and interquartile range from five independent experiments.

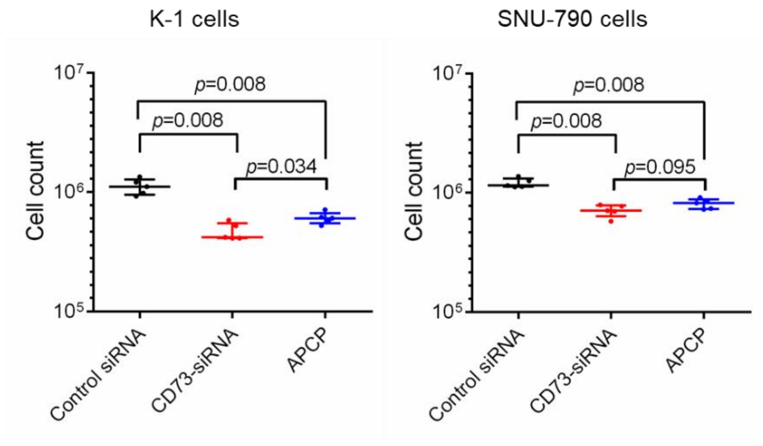

Figure S2. Downregulation of CD73 expression inhibits cell proliferation. Cell proliferation was measured using a cell counting after treatment with CD73-siRNA and APCP in both K-1 and SNU-790 cells. The number of viable cells was counted using a hemocytometer.

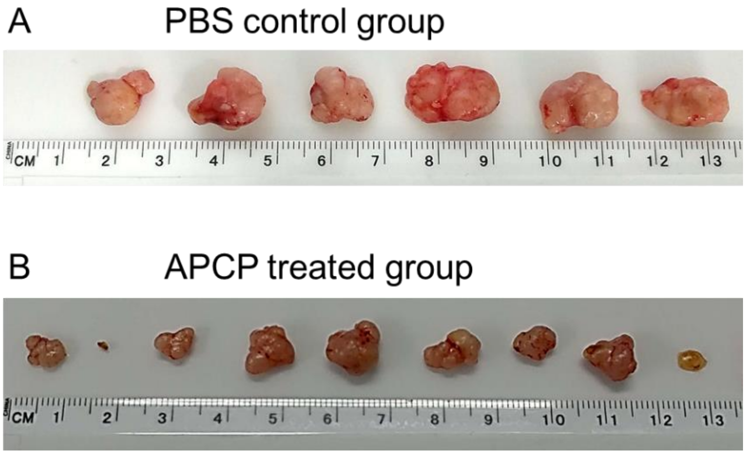

Figure S3. Photographs of gross specimens of tumors in BALB/c nude mice shown in Figure 6A.

Figure 3A and Figure S1A

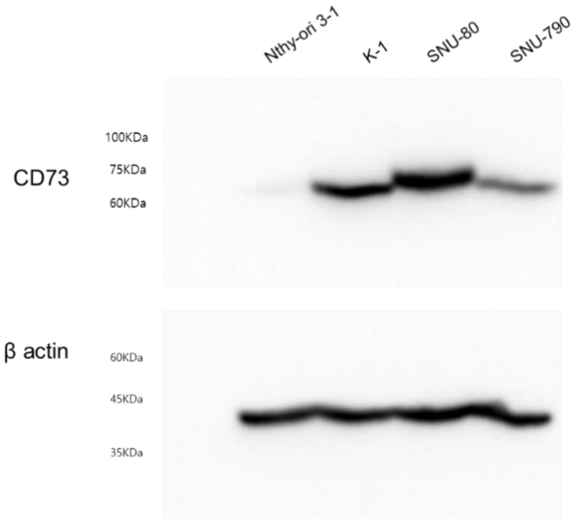

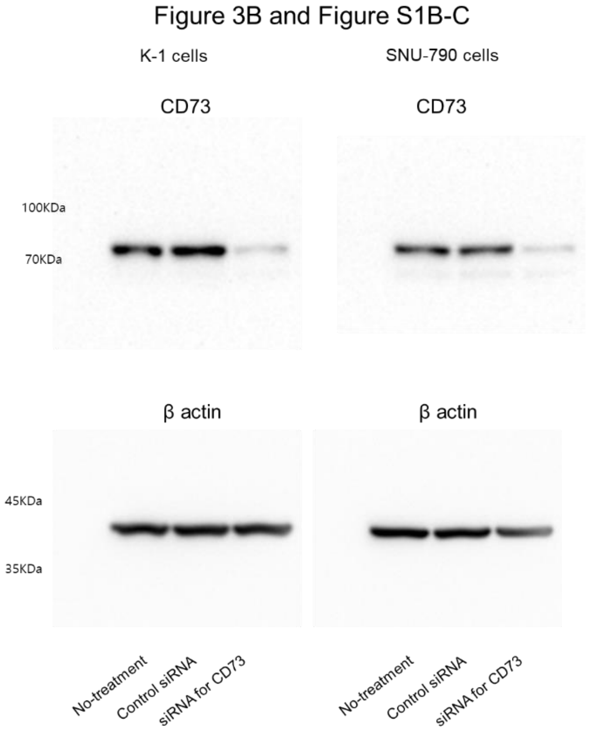

Figure S4. Detail information about Figure 3 and Figure S1.

© 2020 by the authors. Licensee MDPI, Basel, Switzerland. This article is an open access article distributed under the terms and conditions of the Creative Commons Attribution (CC BY) license (http://creativecommons.org/licenses/by/4.0/).
